# Role of Cardiac Magnetic Resonance in Inflammatory and Infiltrative Cardiomyopathies: A Narrative Review

**DOI:** 10.3390/jcm13164733

**Published:** 2024-08-12

**Authors:** Davide Marchetti, Federica Buzzi, Riccardo Di Febo, Sara Modugno, Matteo Schillaci, Pasquale Paolisso, Marco Doldi, Eleonora Melotti, Angelo Ratti, Andrea Provera, Gianluca Guarnieri, Riccardo Terzi, Michele Gallazzi, Edoardo Conte, Daniele Andreini

**Affiliations:** 1 Division of University Cardiology and Cardiac Imaging, IRCCS Ospedale Galeazzi Sant’Ambrogio, 20157 Milan, Italy; davide.marchetti@grupposandonato.it (D.M.);; 2 Department of Clinical Sciences and Community Health, University of Milan, 20122 Milan, Italy; 3 Department of Biomedical and Clinical Sciences, University of Milan, 20122 Milan, Italy

**Keywords:** cardiac magnetic resonance imaging, cardiomiopathy, inflammation, systemic disease, infiltrative diseases, cardiac sarcoidosis, amyloidosis, Anderson–Fabry disease, iron overload

## Abstract

Cardiac magnetic resonance (CMR) has acquired a pivotal role in modern cardiology. It represents the gold standard for biventricular volume and systolic function assessment. Moreover, CMR allows for non-invasive myocardial tissue evaluation, highlighting tissue edema, fibrosis, fibro-fatty infiltration and iron overload. This manuscript aims to review the impact of CMR in the main inflammatory and infiltrative cardiomyopathies, providing details on specific imaging patterns and insights regarding the most relevant trials in the setting.

## 1. Introduction

Cardiac magnetic resonance (CMR) has acquired a pivotal role in modern cardiology. It represents the gold standard for biventricular volume and systolic function assessment [1]. CMR allows for non-invasive myocardial tissue evaluation, highlighting tissue edema, fibrosis, fibro-fatty infiltration and iron overload. Moreover, even if its clinical use is limited by specific contraindications, CMR imaging does not suffer from acoustic window variability, which represents a potential limitation to echocardiography.

According to the latest ESC Guidelines, CMR should be performed (Class I Level B recommendation) in the initial evaluation of cardiomyopathies and should be repeated (class IIa Level B-C recommendation) for disease progression monitoring, risk stratification, and therapeutic response assessment in the setting of inflammatory and infiltrative cardiomyopathies such as cardiac amyloidosis, Anderson–Fabry disease, sarcoidosis and hemochromatosis with cardiac involvement. CMR should be considered to detect early disease in genotype-positive/phenotype-negative family members of patients [2].

## 2. Inflammatory Phenotypes and “Hot Phase” Presentation in Cardiomyopathies

Recently, inflammation has been suggested as the key process in cardiomyopathy pathophysiology [3]. Indeed, acute inflammatory “hot phases” can be recognized among a wide spectrum of cardiomyopathy phenotypes.

Acute inflammatory phases are characterized by myocarditis-like presentation (acute chest pain, dyspnea, palpitations, syncope, heart failure sudden cardiac death) [4], with a dynamic increase in serum cardiac troponins, arrhythmias, myocardial edema on CMR and a higher long-term risk of adverse myocardial remodeling, myocardial fibrosis, heart failure and ventricular arrhythmias [5].

The most relevant and demonstrated pathophysiologic mechanism involved is acute myocardial necrosis and fibrofatty infiltration, rather than gradual apoptosis, especially in desmoplakin (DSP)-mutated patients. However, further investigation is still needed to better understand the triggers of myocardial inflammation [6,7].

Myocardial edema is assessed by T2-weighted (T2w) imaging sequences [8]. Myocardial T1 mapping, T2 mapping and extracellular volume (ECV) have been established as validated and reproducible tools for quantification of focal or diffuse edema. Myocardial T2 mapping is superior to T2w sequences [9,10] for early detection of myocardial injury, preceding symptoms, worsening of ejection fraction and myocardial remodeling in cardiomyopathies [11].

**Figure 1:**
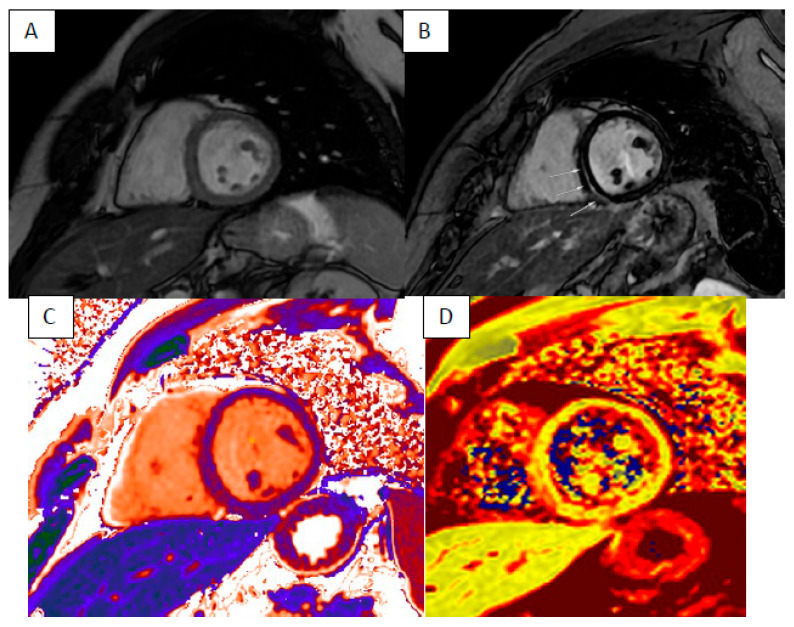
“Hot phase” presentation of NDLVC—short axis cine FIESTA (**A**), short axis LGE sequence showing basal posterior septal intramyocardial enhancement (highlighted with arrows) (**B**) with elevated T1 (**C**) and T2 times (**D**).

**Figure 2:**
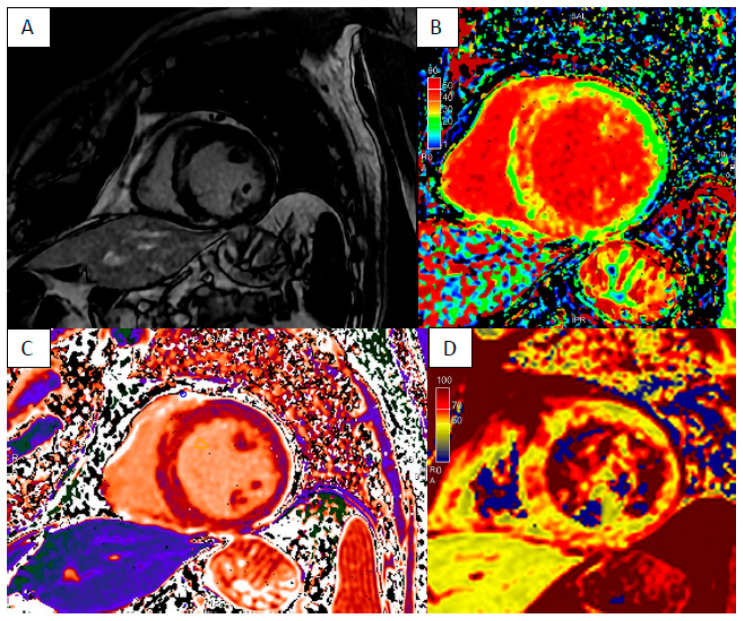
“Hot phase” presentation of DCM—short axis LGE sequence septal intramyocardial enhancement (**A**) with elevated ECM (**B**), T1 (**C**) and T2 times (**D**).

**Figure 3:**
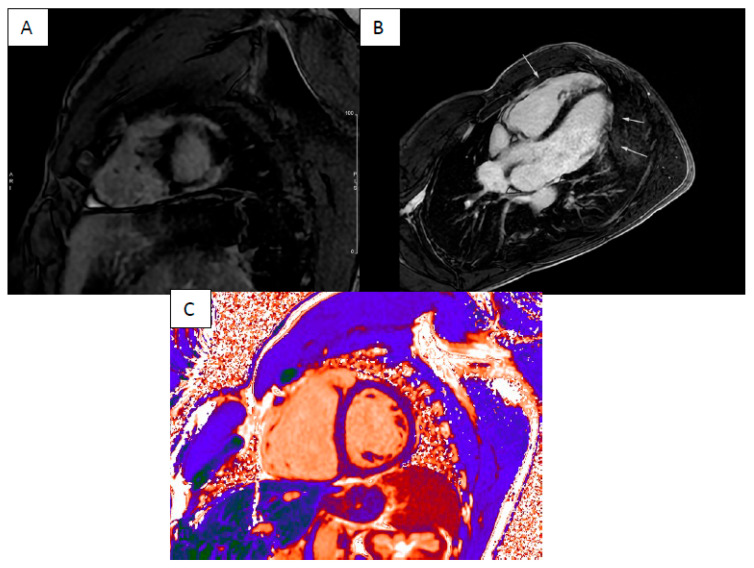
Biventricular ARVC—short axis LGE sequence (**A**) and long axis LGE sequence (**B**) showing diffuse intramyocardial enhancement involving left and right ventricles (LGE highlighted with arrows) with elevated T1 times (**C**).

Arrhythmogenic right ventricular cardiomyopathy (ARVC), dilated cardiomyopathy (DCM) and non-dilated left ventricular cardiomyopathy (NDLVC) can debut with heart failure and ventricular arrhythmias. Differential diagnosis could be difficult due to the complexity and overlap of clinical scenarios, and only approximately half of those cases are correctly diagnosed during the “hot phase” presentation [12]. CMR plays a key role in differential diagnosis because it highlights the specific phenotypes of presentation as ventricular dilatation, segmental or global systolic dysfunction, myocardial fibrosis or fibro-fatty replacement (Figure 1, Figure 2 and Figure 3).

Cardiomyopathies could share an inflammatory background and genetic predisposition with myocarditis [6,13,14]. The quantification of myocardial edema has prognostic role since myocardial T2 relaxation time mapping is associated with left ventricle overload, reverse modeling, quality of life, the 6 min walking test, glomerular filtration rate and N-terminal pro-brain natriuretic peptide (NT-proBNP) in heart failure patients [11,15,16].

Because of the high sensitivity of CMR mapping, the finding of subtle myocardial inflammation could guide the indication to perform an endomyocardial biopsy, enhancing the possibility of a specific diagnosis and treatment [4].

Inflammation could represent a stimulus to phenotypic progression of disease in patients with genetic susceptibility: recently, Sikking et al. suggested that in patients with a P/LP variant of DCM-associated genes, EMB-proven myocardial inflammation was associated with earlier-onset disease [17,18].

**Figure 4:**
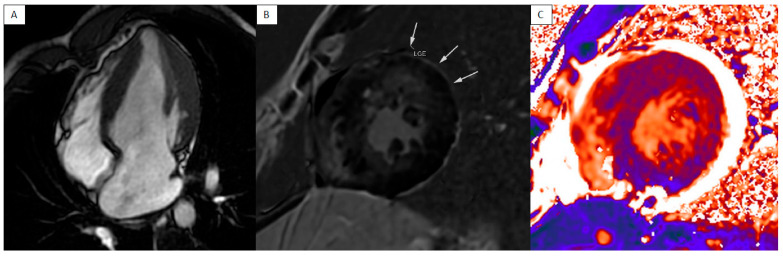
HCM—FIESTA four-chamber view showing LV hypertrophy (**A**) with mild enhancement LGE highlighted with arrows) and elevated T1 times in hypertrophic segments (**B**,**C**).

In hypertrophic cardiomyopathy (HCM), sarcomere dysregulation leads to pro-inflammatory pathway activation, determining a chronic inflammatory state that worsen disease severity and patient prognosis. CMR can play a significative role in individuating myocardial inflammation and fibrosis (Figure 4) [19,20]. Moreover, T1 and T2 relaxation times’ increase may anticipate hypertrophy onset, as demonstrated by Huang L et al. [11,21].

Patients who received a heart transplant should be monitored in dedicated, high-volume centers. Heart-graft rejection is an immunological condition that leads to inflammatory graft damage. Various authors are proposing feature-tracking parameters, T1, T2 and ECV mapping as markers to predict clinical graft rejection in heart-transplant recipients [22].

## 3. Cardiac Sarcoidosis

Sarcoidosis is a systemic inflammatory disease characterized by the formation of non-caseous granuloma in several organs, including the heart. The clinical manifestations of cardiac involvement are conduction abnormalities, ventricular arrhythmias, sudden cardiac death (SCD) and congestive heart failure [23]. Diagnosing cardiac sarcoidosis (CS) remains difficult due to the diverse range of symptoms and the limited sensitivity of non-invasive tests, often necessitating an endomyocardial biopsy for a conclusive diagnosis.

The use of CMR plays a potential role in early diagnosis, risk stratification and monitoring treatment response in CS patients [24]. Smedema et al. followed a cohort of 59 patients with suspected CS and demonstrated that CMR can yield a diagnosis of CS with a sensitivity of 100%, specificity of 78% and overall accuracy of 83%, compared to the modified Japanese guidelines as the gold-standard diagnostic criteria [25].

CMR offers a comprehensive evaluation of biventricular geometry, function and tissue characteristics. It facilitates the identification of pathological myocardial regions, guiding the optimal placement of endomyocardial biopsies. This targeted approach enhances the sensitivity of the invasive procedure, thereby improving diagnostic accuracy. The acute inflammatory phase, characterized by granulomatous lesions and edema, may display non-coronary distribution wall motion abnormalities, increased myocardial wall thickness and increased signal on T2-weighted images.

**Figure 5:**
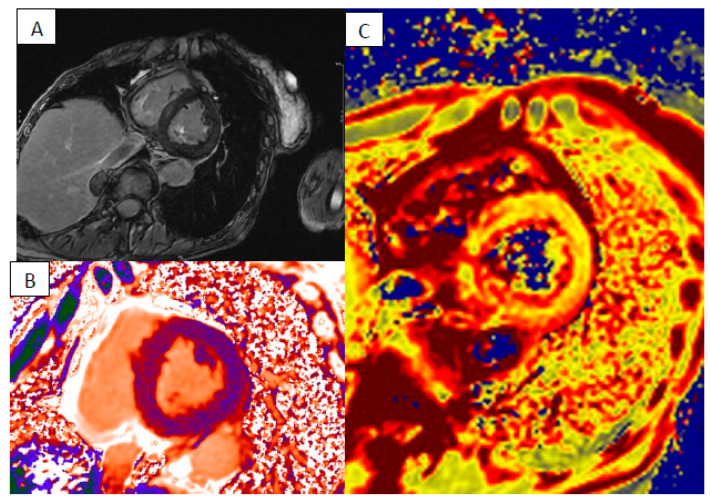
Cardiac sarcoidosis—short axis LGE sequence (**A**) showing focal septal intramyocardial enhancement with elevated T1 times (**B**) and T2 times (**C**).

In chronic stages, the typical CMR findings are myocardial wall thinning and non-ischemic LGE, associated with the absence of myocardial edema assessed by T2w images or T2 mapping (Figure 5) [26].

There is no specific LGE pattern pathognomonic for CS, thus differential diagnosis with other inflammatory diseases can be challenging. The most common pattern is one or more patchy regions of LGE with a non-ischemic distribution, predominantly localized in the basal wall, the lateral wall and septum with subepicardial or midwall involvement. However transmural or subendocardial enhancement in other locations has also been observed [27].

The presence of LGE provides an important value to prognosis and therapeutic management. A systematic review and meta-analysis involving 760 patients with known or suspected sarcoidosis reported that patients with LGE are at increased risk of death from any cause and arrhythmogenic events (ventricular arrhythmia, ICD shock, SCD): odds ratio for all-cause mortality 3.06, *p* < 0.03, odds ratio for composite outcome 10.74, *p* < 0.00001 [28].

In a study by Kouranos et al., 321 patients with extracardiac biopsy-proven sarcoidosis were followed for primary (composite of all-cause mortality, sustained ventricular tachycardia [VT], hospitalization for heart failure) and secondary (non-sustained VT) endpoints. LGE was the only independent predictor of the primary endpoints. Its predictive value was maintained in the subgroup with cardiac symptoms or abnormal electrocardiography findings [24].

According to the 2014 HRS guidelines [29], CS patients with LGE and normal left ventricular ejection fraction (LVEF) may undergo an electrophysiological study for arrhythmic risk stratification. Accumulating clinical evidence from the scientific literature may justify ICD implantation.

Ise et al. studied the correlations between the extent of LGE and the outcomes after steroid therapy in 43 consecutive LGE-positive CS patients. They found that extensive LGE (LGE mass ≥ 20%) predicts higher incidence of adverse outcomes, including cardiac death and hospitalization for heart failure. The extension of LGE is also associated with the absence of LV functional improvement and a decrease of the LV end-diastolic volume index, instead of patients with lower LGE mass (<20% of LV mass) [30].

CS may also affect the right ventricle (RV). A meta-analysis by Wang et al. included eight studies and a total of 899 patients with a mean follow-up duration of 3.2 ± 0.7 years, evaluating the prognostic value of RV alterations on CMR in patients with known or suspected CS. The presence of reduced RV systolic function or RV LGE was significantly associated with adverse outcomes, including all-cause death, adverse cardiovascular events and SCD. Moreover, the presence of RV LGE was a strong independent predictor for SCD, enhancing prognostic stratification over LV LGE involvement [31]: patients with RV LGE had a significant risk for composite events (RR: 4.8, *p* < 0.01) and a higher risk for SCD (RR: 9.5, *p* < 0.01) than patients without RV LGE. In a real-life scenario, however, it must be noted that RV tissue characterization is difficult because the relatively reduced thickness of the myocardial wall may overcome the spatial resolution of CMR.

Considering that LGE better identifies focal rather than diffuse processes, the addition myocardial mapping improves the detection of diffuse myocardial involvement and inflammation. A study by Greulich et al. enrolled patient with CS vs. controls, both groups with preserved LVEF. CMR analysis showed that patients with sarcoidosis, regardless of LGE extension, have significantly higher values of native T1, T2 and extracellular volume compared with those of controls [32].

Moreover, non-invasive CS evaluation may benefit from the emerging attention on hybrid imaging: Greulich at al. investigated the value of hybrid CMR and 18F-fluorodeoxyglucose positron emission tomography (FDG-PET) for detection and differentiation of active from chronic disease. They enrolled 36 patients with biopsy-proven extracardiac sarcoidosis and suspected CS. In 18 patients, they diagnosed CS using CMR with LGE, T1 and T2 mapping and classified CS into active and chronic by simultaneous FDG-PET. Fourteen patients presented LGE, four patients were LGE-negative but demonstrated increased mapping values. These results support the role of T1 mapping in the evaluation of CS, as it allowed the detection of four additional CS patients that would have been missed using only LGE. Among these four CS patients, three were PET-positive, indicating active CS [33].

CMR holds promise not only for diagnosis but also for patient monitoring and follow-up post medical therapy. Puntmann et al. studied 53 patients with biopsy-proven extracardiac sarcoidosis by CMR and proposed a follow-up sub-study in 40 of them (mean follow-up interval: 144 ± 35 days). Eighteen patients underwent anti-inflammatory treatment for systemic symptoms. The authors found higher myocardial native T1 and T2 in patients when compared with those in controls and observed a significant reduction of native T1 and T2 values in the patients who underwent treatment [34].

These studies underscore the possible additional value of T1 and T2 mapping in the diagnosis of CS as they offer better detection of inflammation (Table 1), disease activity and response to immunosuppressive therapy than LGE alone, which does not differentiate acute from chronic phases. Potentially reversible myocardial changes, detected by mapping analysis, might precede irreversible fibrosis, detected by LGE. However, considering that LGE-negative CS patients have good prognosis, clinical relevance of abnormal T1 and T2 mapping values remains still unclear [32].

## 4. Cardiac Amyloidosis

**Figure 6:**
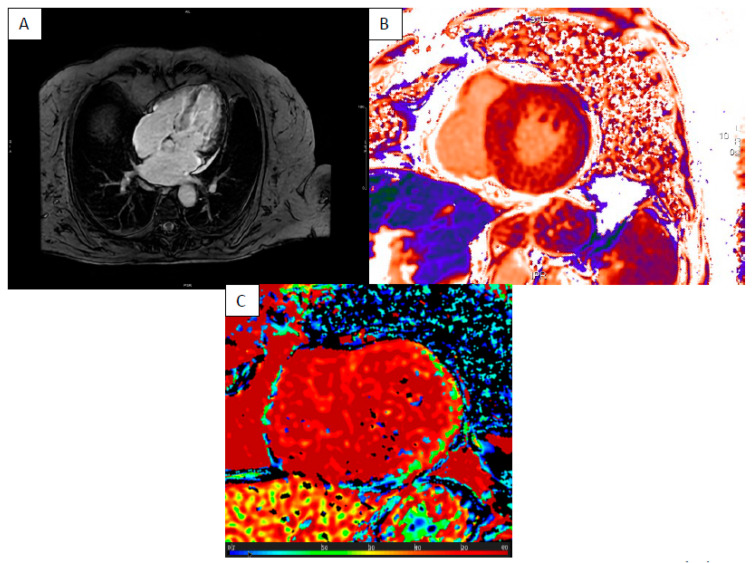
Cardiac amyloidosis—long axis LGE sequence (**A**) showing diffuse sub-epicardial enhancement involving the atria and left and right ventricles with diffuse markedly elevated T1 times (**B**) and ECV (**C**).

Cardiac amyloidosis (CA) is an infiltrative cardiomyopathy characterized by amyloid protein deposition in the myocardial extracellular space, leading to myocardial hypertrophy and dysfunction progressing to heart failure (Figure 6).

Among more than thirty different types of amyloid protein involved, those more likely to be associated with cardiac involvement are misfolded immunoglobulin light chain (light-chain amyloidosis—AL) and transthyretin (transthyretin amyloidosis—ATTR).

The diagnostic algorithm is based on multimodality non-invasive imaging. Clinical suspicion often derives from epidemiologic features, clinical criteria and echocardiographic typical “red flags” [35].

The initial evaluation includes 9mTc pyrophosphate (PYP), 3,3-diphosphono-1,2-propanedicarboxylic acid (DPD) and hydroxymethylene diphosphonate (HMDP) bone scintigraphy and blood/urine protein assessment and immunofixation. Endomyocardial biopsy is reserved for patients with discordant clinical and imaging findings or uncertain imaging results.

According to the ESC 2021 Position Statement on Diagnosis and Treatment of Cardiac Amyloidosis, CMR is needed especially when scintigraphy is negative, with or without positive hematologic tests [35].

CA can manifest across a wide spectrum of morphological phenotypes. The most common presentation is concentric left ventricular hypertrophy (LVH), although eccentric LVH, concentric remodeling and normal geometry have also been observed [36]. Additionally, these phenotypes may be accompanied by right ventricular hypertrophy [37]. Left ventricular ejection fraction (LVEF) typically remains preserved or only mildly reduced, primarily due to a concurrent decrease in left ventricular end-diastolic volume. Consequently, stroke volume (SV) emerges as a more sensitive parameter for assessing systolic dysfunction, as well as for gauging amyloid burden in early stages, tracking disease progression over time and predicting prognosis [38].

Global longitudinal strain (GLS) has proved to be superior to LVEF in diagnosis and prognosis stratification [39,40]. CA shows a specific “apical sparing” GLS pattern that could be identified through CMR feature tracking (FT) to provide differential diagnosis in early detection of cardiac involvement in systemic disease, uncertain diagnosis and for risk stratification, offering incremental prognostic value over current prognostic evaluation [41].

Right ventricular (RV) dysfunction is a strong predictor of poor outcomes in CA. Fine et al. investigated a cohort of 93 CA patients for 26 months (median) and found that RV function expressed by RV free wall strain was strongly associated with cardiovascular outcomes [41]. A similar study by Bodez et al. demonstrated that low TAPSE independently predicted major adverse cardiac events (MACE), defined as death, heart transplantation and acute heart failure [42].

The utilization of late gadolinium enhancement (LGE) sequences is of significant clinical and research interest in these patients, as they effectively demonstrate amyloid myocardial infiltration. Gadolinium kinetics is altered by amyloid protein in the myocardium, leading to a difficult selection of inversion recovery time for traditional LGE imaging. Phase-sensitive inversion recovery sequences (PSIR) are highly sensible and specific to assessing a diffuse LGE pattern [43,44].

A typical amyloid deposition pattern is shown by patchy LGE to diffuse (subendocardial or transmural) LGE in a non-coronary distribution [45].

T1 relaxation time reflects intracellular and interstitial composition [46,47]. Native T1 relaxation time increases in CA due to amyloid deposition, myocardial fibrosis and edema. T1 mapping values are usually higher than HCM and other phenocopies, offering a valid tool for differential diagnosis [48].

In the last decade, myocardial mapping has demonstrated high accuracy in detecting early cardiac involvement before LGE and superiority to GLS in differential diagnosis with HCM, correlation with systolic and diastolic dysfunction parameters, reliability for assessment of regression of amyloid burden during therapy and correlation with mortality during follow-up [49,50].

A recent meta-analysis by Boretto et al. including 2199 patients and 19 studies outlined that both left heart tissue characterization parameters such as elevated extracellular volume (HR 3.95), extension of LV LGE (HR 2.69), elevated native T1 (HR 2.19) and functional parameters such as reduced LV GLS (HR 1.91) and reduced LV EF (HR 1.20) were associated with increased all-cause mortality [37,51].

Coronary microvasculature is impaired due to amyloid infiltration, causing severe lumen reduction and severe reduction in capillary density. Chacko et al. demonstrated that CMR stress perfusion can highlight inducible myocardial ischemia in CA that correlates with amyloid burden [52].

In CA, misfolded proteins deposit in the interstitial space, therefore, measuring ECV does give an accurate estimation of CA burden [53]. ECV has demonstrated better diagnostic and prognostic performance over T1 mapping [54], and LGE correlates with the functional state assessed through the 6 min walking test, cardiac biomarkers and disease severity [55,56], making ECV an ideal marker for regression of pathology during CA treatment [57]. The toxic effect of myocardial amyloid deposition causes myocardial edema and elevation of T2 relaxation times, even if no correlation is found between edema assessed on EMB and T2 relaxation times [58]. Some authors argue that myocardial T2 relaxation time tends to be higher in AL amyloidosis, probably due to the greater toxicity of light chain amyloid, and then may be useful to discriminate between ATTR and AL cardiac amyloidosis [53].

## 5. Anderson–Fabry Disease

Anderson–Fabry disease (AFD) is a rare X-linked disorder caused by a deficiency in the alpha-galactosidase (α-Gal) enzyme, which is encoded by the Alpha Galactosidase gene (GLA). As a result, there is a progressive accumulation of glycosphingolipids in many cell types and tissues, including the heart. Glycosphingolipid overload leads to cellular and vascular dysfunction through inflammatory and neurohormonal mechanisms, consequently leading to tissue ischemia, hypertrophy and fibrosis [59,60].

The common course of cardiac changes in AFD that begin in childhood consist of a pre-clinical accumulation phase. Initially, patients exhibit normal left ventricular mass, but over time, they accumulate glycosphingolipids within myocytes. Later, progressive accumulation causes inflammatory changes and reactive hypertrophy, leading to concentric LVH and myocardial fibrosis with a preferential basal inferolateral wall location. Finally, tissue damage results in fibrosis and deterioration of myocardial function [61].

Cardiac involvement in AFD is common and is the leading cause of morbidity and mortality due to the development of heart failure and ventricular arrhythmias [59].

**Figure 7:**
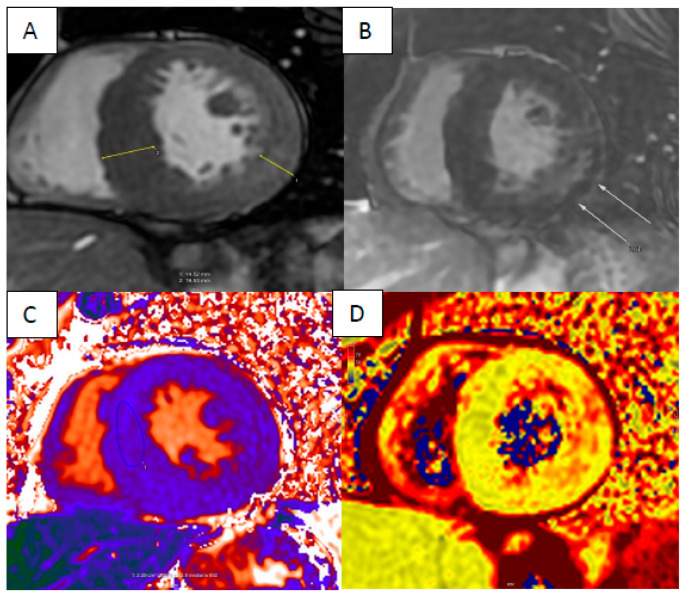
Anderson–Fabry disease—short axis FIESTA sequence showing concentric hypertrophy (**A**), typical basal inferolateral intramyocardial enhancement (LGE highlighted with arrows) (**B**) with markedly reduced T1 times (**C**) and elevated T2 times in inferolateral segments (**D**).

CMR is a primary non-invasive and multi-parametric imaging technique used to evaluate cardiac involvement in AFD (Figure 7).

Based on a recent expert consensus document, it is recommended that CMR should be performed in all adult patients [62]. The primary CMR finding in patients with AFD is concentric LVH, involving papillary muscles and trabeculations [63]. LVH has been found to correlate with α-galactosidase activity levels, therefore, females are less likely to develop LVH, and it evolves about 10 years later than in males.

In more advanced stages, when fibrosis is present, hypertrophy may become asymmetric with septal thickening and inferolateral wall fibrotic thinning. Right ventricular hypertrophy is also common and may progress to right ventricular dysfunction and dilation [64].

Myocardial mapping evaluation by CMR is crucial in AFD. In the early stages of the disease, even before the development of LVH, the overload of glycosphingolipids leads to a decrease in native T1 values [61]. In a study conducted on sixty-three patients with confirmed AFD, Pica et al. reported reduced native T1 in 89% of AFD patients with LVH (853 ± 50 ms), but also in 48% of those without LVH (904 ± 46 ms), compared to that in healthy volunteers (968 ± 32 ms). T1 reduction before the onset of LVH is associated with echocardiographic parameters of cardiac dysfunction, suggesting that a low T1 detects early cardiac involvement [65].

Ponsiglione et al. carried out a meta-analysis in which they compared native T1 values of AFD patients to those of healthy controls. The results showed a reduction in T1 values in AFD patients (984 ± 47 ms in AFD patients vs. 1016 ± 26 ms in controls). Moreover, in AFD patients, there was an inverse correlation between native T1 values and male gender and LVH. The influence of gender on native T1 values is explained by the fact that in heterozygous women, the activity of the α-Gal enzyme is partially maintained, resulting in mild to absent accumulation of lipids in myocytes. In the late hypertrophic stages of AFD, inflammation and interstitial fibrosis develop, leading to pseudo-normalization or elevation of native T1 times [66]. Septal T1 values usually remain low, while inferolateral values, where fibrosis is mainly localized, may be increased [65]. In patients with LVH, preserved myocardial inhomogeneity of T1 values could help differentiate AFD from other common causes of LVH (phenocopies).

Camporeale et al. enrolled 44 AFD patients without LVH and demonstrated that the presence of low T1 values is associated with an increased risk of disease worsening at 12-month follow-up. This finding suggests that T1 values may be a potential new marker for prognostic stratification and guiding therapeutic approaches in AFD [67].

In the early stages of AFD, the intracellular accumulation of glycosphingolipids leads to a reduction in interstitial spaces. As a result, ECV is typically normal or even decreased. The role of ECV is limited to more advanced stages of the disease when fibrosis is diffuse [68].

LGE is present in almost half of AFD patients and it is usually localized in the basal and mid-inferior–lateral wall of the left ventricle, with mid-wall or sub-epicardial involvement. This distribution may be due to inhomogeneous left ventricle wall stress, but the true cause remains uncertain [69]. Several studies investigated the association between CMR findings and outcomes in AFD. In a retrospective cohort study, Hanneman et al. evaluated 90 patients after a follow-up period of 3.6 years. LGE and LVH were the only independent predictors of a composite end point of arrhythmia, heart failure and cardiac death [70]. Earlier studies reported that LGE reflects fibrosis, but the latest reports suggest that LGE may represent chronic inflammation in AFD. Augusto et al. conducted a prospective international multicenter study involving 186 AFD patients, 58 patients with other cardiovascular diseases (HCM or chronic coronary syndrome) and 59 healthy volunteers. They found that subjects with LGE in the basal inferolateral wall (BIFL) had significantly higher segmental and global T2 values compared to those in patients without LGE. Interestingly, the elevation of T2 in LGE was higher in AFD patients compared to that of other cardiovascular diseases. The regional T2 increase was strongly associated with elevated troponin. After a year, both T2 and troponin elevations were chronic, and a BIFL T2 increase was associated with clinical worsening. These findings indicate that patients with AFD exhibit chronic myocardial edema that is closely linked to cardiac injury, and this association plays a prognostic role [71].

AFD is also characterized by endothelial storage and dysfunction leading to abnormalities in microvascular perfusion. Knott et al. found that stress CMR perfusion mapping (MBF) highlights impaired myocardial blood flow in AFD patients compared with healthy volunteers, even in the absence of LVH. Reduced stress MBF is also related to disease severity as it correlates with wall thickness, inflammation (elevated T2) and scar (elevated ECV and LGE) [72].

CMR may also help in detecting early cardiac involvement in AFD through the assessment of myocardial strain. Mathur et al. reported that AFD patients have a significantly lower base-to-apex circumferential strain (CS) gradient compared to that of healthy controls. This value differentiates AFD patients with no LVH or LGE from healthy subjects, regardless of T1 values. On the contrary, the authors did not find a difference in global longitudinal strain, global circumferential strain and base-to-apex longitudinal strain [73].

## 6. Myocardial Iron Overload

Excessive accumulation of iron in the myocardium is a notable contributor to heart failure and can result from various factors. These include genetic disorders like hereditary hemochromatosis, or prolonged transfusion therapy, as seen in conditions such as thalassemia or sickle cell disease.

The clinical manifestations of iron deposition can vary. Patients may not experience any symptoms in the early stages of the disease, but once symptoms of cardiac failure develop, there is a rapid decline in their clinical condition. Medical interventions do not often yield significant improvements. Initially, patients can present exertional dyspnea due to diastolic dysfunction and, as the disease progresses, it leads to dilated cardiomyopathy with impaired left ventricular function and fibrosis.

CMR can quantify myocardial iron overload with T2* relaxation measurement [74]. Iron deposition in the myocardium has a significant impact on the myocardial MR signal, affecting both transverse and longitudinal relaxation. Intracellular, sarcoplasmatic iron particles act like tiny magnets that disrupt the local magnetic field and cause neighboring protons to lose synchronization, resulting in reduced T2* and T2 relaxation times. The paramagnetic interaction also leads to the transfer of energy from the spin system to the surrounding tissue, thereby affecting longitudinal relaxation.

T1 relaxometry could be used to investigate whether different forms of iron have varying effects on transverse and longitudinal relaxation, and whether this information has clinical relevance. In cases of mild iron overload, caution should be exercised when using T2* measurements because other factors may influence the homogeneity of the magnetic field [75]. T2 and T1 measurements may be a more suitable option because they are less influenced by local field inhomogeneity. However, the use of quantitative cardiovascular MR relaxometry has not met all expectations due to the variability in these parameters in vivo. This variability is attributed to the complex structure of the myocardium, to issues with respiratory and cardiac motion and to the presence of blood flow.

Efforts are being made to improve the accuracy and reproducibility of T2 and T1 measurements in the myocardium. Advanced techniques, such as motion correction and respiratory gating, are being developed to minimize the impact of motion artifacts. In addition, the use of higher field strengths and more sophisticated imaging sequences may further enhance the reliability of quantitative relaxometry measurements in clinical practice.

As iron accumulates in the heart in its normal storage form, the T2* value decreases. However, cardiac function remains largely unaffected until a certain threshold is reached. At this point, when the iron storage capacity is exceeded, non-transferrin-bound iron (NTBI) begins to deposit, causing significant damage to the heart due to its toxic and inflammatory effects [76]. Thus, the relationship between measured T2* and cardiac function is variable until a critical level is reached, after which rapid deterioration occurs. This explains why the identification of abnormal systolic function is a late sign of iron toxicity.

Iron is removed more slowly from the heart than from the liver, which may contribute to the high mortality of patients with established cardiomyopathy despite intensive iron chelation. Using the T2* technique, it is possible to identify patients who require intensive chelation much earlier prior to the onset of systolic dysfunction, which should avoid the mortality associated with overt heart failure [77].

Brendel et al. conducted a study involving 614 patients who underwent CMR for various clinical indications. A single midventricular short axis was used for T2* mapping as part of a comprehensive routine CMR protocol. The degree of cardiac iron overload was categorized as mild (15 ms < T2* < 20 ms), moderate (10 ms < T2* < 15 ms) or severe (T2* < 10 ms). The degree of hepatic iron overload was characterized as mild (4 ms < T2* < 8 ms), moderate (2 ms < T2* < 4 ms) or severe (T2* < 2 ms). The T2* maps revealed incidental cardiac iron overload in 1.1% of patients, while liver iron overload was found in 3.6% of patients. There was no significant correlation between LVEF and T2*. The main benefit of standardized T2* analysis is the early detection of myocardial iron overload, which cannot be predicted by laboratory iron values. This data could allow for early initiation of treatment before the onset of symptoms, as conventional assessment of cardiac function may only detect advanced disease [78].

The working group of Ojha, focusing solely on patients with thalassemia, observed no significant correlation between left ventricular ejection fraction (LVEF) and T2* values. However, they did find a notable correlation between T2* values and left ventricular strain values [79]. This underscores the notion that T2* imaging can effectively detect early-stage disease, thereby enhancing the identification of suitable candidates for treatment and improving prognosis stratification.

In a prospective study by Sado et al., patients suspected of having iron overload and healthy volunteers were recruited and referred for clinical MRI assessment of myocardial iron. The purpose of the study was to demonstrate that myocardial T1 mapping can be used as an alternative method for quantifying cardiac iron. The researchers found that there was a good overall linear correlation between T1 and T2* measurements, especially in patients with a low T2* value. Interestingly, one-third of the patients with a normal T2* value had a low T1 value. The study also found that neither left ventricular ejection fraction nor indexed left ventricular end-diastolic volume correlated with T2* or T1 measurements. There are numerous advantages to utilizing T1 measurements. They are more straightforward to use in clinical settings, demand less offline analysis and time and exhibit greater sensitivity compared to T2* evaluation. Additionally, T1 measurements boast higher reproducibility, enabling more precise monitoring of disease progression [80].

Saadatifar et al. conducted a study to evaluate the effectiveness of biochemical markers and cardiac T2* MRI in diagnosing cardiac iron overload. The study included 62 thalassemia patients who were analyzed for blood markers such as ferritin, copeptin, hs-CRP and NT-proBNP. The results showed that 25.8% of the patients had cardiac iron overload based on T2* MRI, which was characterized by a T2* value of less than 20 ms. Interestingly, the study found that most of the inflammatory factors failed to diagnose early cardiac iron overload. This highlights the importance of cardiac MRI, which is considered the gold standard tool for diagnosing cardiac siderosis in such cases [81].

## 7. Limitation of Cardiac MRI

Although CMR is a key imaging investigation in the setting of cardiomyopathies, it must be acknowledged that it suffers from some limitations.

CMR imaging demands high compliance from the patient, who must remain still to avoid motion artifacts and must hold their breath as required for the entire duration of the exam, which usually lasts approximately one hour. Patients who are clinically unstable, who have a respiratory condition or even who have a language barrier that could limit their compliance should be considered unsuitable.

Metallic devices are an issue for MR imaging because they must be assessed for compatibility before patient candidacy for CMR. Even in the case of compatibility, metallic artifacts could affect image quality.

Nowadays, local availability and costs are still a key limitation because CMR is expensive and rather difficult to provide.

The role of a multimodal imaging physician specialized in cardiovascular imaging is becoming increasingly vital. Such a professional possesses dedicated expertise in various imaging techniques and can adeptly select the most suitable investigation based on diagnostic suspicion, patient characteristics and the availability of diagnostic resources within the hospital and local area.

## 8. Conclusions

Cardiac MR is a prominent imaging technique for diagnosis, risk stratification and therapeutic monitoring in the setting of infiltrative and inflammatory cardiomyopathies.

The pivotal role of CMR in the quantification of heart volume and systolic function, myocardial composition and fibrosis is a determinant factor for modern cardiology.

Inflammatory and infiltrative cardiomyopathies require a multidisciplinary approach where imaging cardiologists, heart failure cardiologists and other physicians must work together.

## Figures and Tables

**Table 1:** CMR features of the major inflammatory cardiomyopathies.

	LV Systolic Function	LV Diastolic Volume	LV Hypertrophy	LGE Pattern	T1 Mapping	T2 Mapping	ECV	T2*
Cardiomiopathy Hot Phase	normal/reduced	normal/dilated	variable	specific pattern	elevated	elevated	elevated	normal
Amyloidosis	normal	normal	Concentric Hypertophy	diffuse	elevated	normal/elevated	elevated	normal
Sarcoidosis	normal/reduced	normal/dilated	not present	patchy	elevated	elevated	elevated	normal
Anderson–Fabry	normal	normal	Concentric Hypertrophy	inferolateral	reduced	elevated	normal	normal
Iron overload	normal/reduced	normal/dilated	not present	mild/absent	reduced	normal/elevated	normal/reduced	reduced
